# Protein-based double-network hydrogels mimicking oral mucosa

**DOI:** 10.3389/fchem.2025.1618870

**Published:** 2025-06-13

**Authors:** Yu Zhang, Liang Dong, Keqing Wang, Yuanqi Cheng, Tian Gao, Jiapeng Yang, Xiaochen Shen, Yi Cao, Bin Xue

**Affiliations:** ^1^ Collaborative Innovation Center of Advanced Microstructures, National Laboratory of Solid State Microstructure, Department of Physics, Nanjing University, Nanjing, China; ^2^ Jinan Microecological Biomedicine Shandong Laboratory, Jinan, China; ^3^ China Tobacco Jiangsu Industrial Co., Ltd., Nanjing, China

**Keywords:** protein hydrogel, double-network hydrogel, oral mucosa, biomimetic material, elastin-like polypeptide

## Abstract

The oral mucosa plays a critical role in protecting the body from external threats and serves as a key site for drug absorption. However, ethical concerns and the high costs associated with animal models traditionally used for oral mucosa research have increased the demand for reliable alternatives. In this study, we developed two types of protein-based double-network hydrogels to replicate the mechanical and structural properties of buccal mucosa and hard palate, respectively. By incorporating polyprotein into the rigid network and elastin-like peptides as the loose network, we fabricated hydrogels that closely resemble the physical properties of natural oral mucosa. These hydrogels exhibit a microporous structure, as well as surface and mechanical properties, and particle permeability comparable to native tissue, while maintaining excellent biocompatibility. We anticipate that these hydrogels can serve as model systems for investigating drug delivery, pathogen interactions, and aerosol particle adsorption in the oral mucosa. The design principles presented in this study could also be extended to fabricate protein-based biomaterials that mimic mucosal tissues in the respiratory, gastrointestinal, and urogenital tracts, providing a general approach for developing biomimetic materials for mucosal tissues.

## 1 Introduction

The oral mucosa constitutes a critical biological interface that not only provides mechanical protection but also serves as an immunological barrier against pathogens, chemical irritants, and allergens (Presland and Jurevic, 2002; Hearnden et al., 2012). Its distinctive characteristics - combining high permeability with dense vascularization - have established it as an optimal platform for transmucosal drug delivery, enabling rapid systemic absorption through sublingual formulations while bypassing gastrointestinal degradation and first-pass hepatic metabolism (Pather et al., 2008; Patel et al., 2011; Lam et al., 2014). Despite these pharmacological advantages, research on mucosal interfaces has long relied on animal models, primarily due to limited availability of viable human tissues and persistent ethical dilemmas (Dutzan et al., 2018; Gaffen and Moutsopoulos, 2020; Zhang et al., 2021). Although animal-derived tissues provide essential biological insights, their application faces critical limitations including significant species-specific differences, technical complexity in experimental manipulation, and growing ethical restrictions. This research paradigm is currently undergoing transformation, driven by evolving regulatory frameworks such as the FDA Modernization Act 2.0 that promotes alternative testing methodologies, coupled with institutional commitments to the 3Rs principles (Replacement, Reduction, Refinement) (Directive, 2002; Adashi et al., 2023). These collective challenges highlight the pressing demand for advanced biomimetic materials capable of precisely replicating both the structural integrity and physiological functionality of native oral mucosa for experimental investigations.

Hydrogels have emerged as ideal candidates for biomimetic materials due to their high water content, structural similarity to biological tissues, excellent biocompatibility, customizable performance, and transparency (Luo et al., 2019; Wei et al., 2024; Zhang et al., 2024). Recent developments in synthetic hydrogels incorporating biomolecules such as proteins (Banta et al., 2010; Li et al., 2016; Wang et al., 2018), peptides (Li and Cao, 2018; Gao et al., 2020), DNAs/RNAs (Wenger et al., 2007; Shao et al., 2017; Wang et al., 2017; Ding et al., 2020), and polysaccharides (Xing et al., 2019; Shokrani et al., 2022) have further expanded their potential by integrating specific biological or physical cues. Among these biomolecule-based systems, protein hydrogels have gained particular attention owing to their uniquely customizable biochemical and mechanical properties (Feig et al., 2018; Lei et al., 2020; Gu et al., 2022; Han et al., 2022; He et al., 2022; Li et al., 2022; Tian et al., 2022; Wu et al., 2022; Yu et al., 2022; Sun et al., 2024).

To date, several effective strategies have been developed, both by our team and others, to rationally design protein hydrogels that mimic the mechanical properties of muscle or cartilage (Lv et al., 2010; Sun et al., 2020; Fu et al., 2023; Yin et al., 2024). These strategies include the fabrication of modularized crosslinking or bearing proteins, protein entanglements, and others. Despite these advances, the application of protein hydrogels to replicate the mechanical properties of the oral mucosa remains relatively unexplored. Given the unique structural and mechanical demands of the oral mucosa, we believe that protein hydrogels specifically designed to mimic these properties could offer significant potential. By leveraging the customizable nature of protein-based materials and synthetic biology (Wu et al., 2018), these hydrogels could be optimized to replicate the soft, yet resilient mechanical properties of oral mucosa, enabling more accurate models for mucosal research and advancing the development of targeted drug delivery systems.

In this study, we developed two types of double-network protein hydrogels to replicate the oral mucosa across different anatomical regions. By incorporating polyproteins into the rigid network and elastin-like polypeptide into the loose network, we created entangled protein networks that mimic the elastic fiber structure characteristic of the oral mucosa. These hydrogels exhibit a microporous structure, surface properties, bulk mechanical properties, and particle permeability comparable to native oral mucosal tissues, while maintaining excellent biocompatibility. We anticipate that these protein-based hydrogels will provide a reliable, cost-effective, and reproducible alternative for research involving mucosal interactions and drug delivery. Furthermore, the design principles established in this study could be applied to other soft tissues, offering a general approach for developing biomimetic materials for tissue engineering and regenerative medicine.

## 2 Materials and methods

### 2.1 Protein engineering

Using standard molecular biology techniques, the twelveploid ELP sequence was constructed by sequentially concatenating ELP monomeric sequences through iterative restriction enzyme digestion and DNA ligase-mediated assembly. For the cGEGc (Cys-GB1-ELP-GB1-Cys) construct, a dimeric ELP sequence was first inserted between two GB1 domains via restriction enzyme and DNA ligase-based assembly, followed by the introduction of cysteine residues at both termini of the resulting sequence. The final multimeric sequences were cloned into the pQE80L vector between BamHI and KpnI sites, followed by transformation into *E. coli* BL21 for protein expression. All plasmid constructs were verified by commercial DNA sequencing to ensure sequence fidelity. The complete amino acid sequences of ELP and cGEGc are provided in Supplementary Material.

### 2.2 Protein purification

The transformed *E. coli* BL21 cultures were grown at 37°C until OD600 reached 0.6–0.8, followed by induction with 1 mM IPTG at 20°C for 16 h. Proteins were purified using Co^2+^ affinity chromatography, yielding approximately 50 mg of protein per liter of culture. While not experimentally tested, Ni^2+^ resin could likely substitute for Co^2+^ in the purification process. Purified proteins were dialyzed in deionized water, freeze-dried, and analyzed by SDS-PAGE, which confirmed their purity (Supplementary Figure S1A). The GB1 domains were validated to adopt correctly folded secondary structures via circular dichroism spectroscopy (Supplementary Figure S1B).

### 2.3 Preparation of oral mucosal samples

Oral mucosa samples were collected from the corresponding regions of the oral cavity of freshly slaughtered pigs. Specifically, buccal mucosa was obtained from the posterior area of the oral commissure, and hard palate mucosa was sampled from the hard, bony area at the roof of the mouth. Multiple specimens were systematically collected from adjacent sites within these predefined anatomical regions to minimize biological variability and ensure sample consistency. The specimens were prepared as 10 mm × 10 mm sections without any preservative treatment and were directly subjected to testing.

All animal studies were carried out in compliance with the regulations and guidelines of the Science and Technology Ethics Committee of Nanjing University, and adhered to the Institutional Animal Care and Use Committee guidelines (IACUC-D2310006).

### 2.4 Hydrogel preparation and mechanical test

To prepare Palatal Gel, 40 mg/mL four-arm PEG-Mal was mixed with 80 mg/mL cGEGc protein at a molar ratio of 1:2. The mixture was then left at room temperature for 2 h to allow complete crosslinking. And 150 mg/mL acrylamide was mixed with 80 mg/mL cGEGc protein and 0.25 mg/mL LAP (lithium phenyl-2,4,6-trimethylbenzoylphosphinate), 3 mg/mL bis-acrylamide, then exposed to UV light for 30 min to prepare Buccal Gel. To enhance the biomimetic properties, 20 mg/mL of ELP protein was added during the pre-mixing stage. The gel was incubated in PBS at 37°C for at least 24 h post-polymerization (with the solution replaced at least once during this period) to induce secondary physical entanglement networks within the primary chemical network.

At 37°C, uniaxial single-stretch; stripping test and cyclic stretching tests were performed using an Instron-5944 tensiometer equipped with a 10 N static load cell. All mechanical testing samples were maintained at dimensions of 10 × 10 × 1 mm. Each experiment was conducted three times, and the average values were reported. Notably, due to the inclusion of physical entanglements, none of the mucosal materials underwent swelling in ultrapure water.

### 2.5 The diffusion test of fluorescent particles with different particle sizes

Fluorescent polystyrene nanoparticles (1 μm, 3 μm, 5 μm, purchased from Ruige Biotechnology Co., Ltd.) were dispersed in phosphate-buffered saline (PBS) at a concentration of 1 mg/mL and sonicated for 10 min to ensure a homogeneous suspension. Hydrogels (10 × 10 × 1 mm) or tissue samples (10 × 10 mm) were placed on a substrate with one side adhered to the surface, ensuring that the bottom surface remained sealed and did not come into direct contact with the fluorescent particles. Using an aerosolizer, the suspension of fluorescent particles was sprayed onto the exposed top surface of the hydrogel or tissue samples to simulate particle deposition. The samples were then incubated at 37°C for 8 h to facilitate particle diffusion. At hourly intervals, the samples were removed and imaged using a fluorescence microscope, with a 1 mm × 1 mm region at the center of each sample selected for data acquisition to avoid edge effects caused by side leakage. Fluorescence imaging was performed with excitation at 488 nm and emission detected between 510 and 550 nm. Fluorescence intensity profiles were quantified using ImageJ software.

Assume that at t = 0, all particles are located on the material surface (defined as z = 0 position), with the downward direction as positive. After time t = T, according to the solution of the one-dimensional diffusion model under the reflective boundary condition, the probability density function describing the particle distribution along the z-direction is given by:

$$P\left(z,T\right)=\frac{1}{\sqrt{\pi DT}}e^{-\frac{z^{2}}{4DT}}$$

where D represents the diffusion coefficient. By fitting the experimentally obtained particle distribution using this equation, the corresponding diffusion coefficient can be determined.

## 3 Results

### 3.1 Design of protein-based double-network hydrogels mimicking oral mucosa

The oral mucosa exhibits distinct structural and compositional variations influenced by its anatomical location, adapting to diverse mechanical demands encountered during physiological functions (Boucher, 2004; Choi et al., 2020a). For example, the masticatory mucosa (e.g., hard palate and gingiva) features a keratinized epithelium anchored to collagen-rich connective tissue, providing durability to withstand chewing forces. In contrast, the lining mucosa (e.g., buccal and sublingual regions) has a non-keratinized epithelium supported by elastic connective tissue, enabling flexibility for dynamic oral movements. To mimic the diverse mechanical demands of oral mucosal tissues, we engineered two distinct protein hydrogels tailored to replicate the unique characteristics of the masticatory mucosa and lining mucosa. These designs leverage the inherent properties of elastin-like polypeptide (ELP) integrated within synthetic polymer networks, enabling the biomimetic replication of tissue mechanical properties through systematic screening of crosslinking parameters. By precisely modulating crosslink density and optimizing the ratio between primary/secondary network components, we achieved precise matching of hydrogel mechanics to native tissue benchmarks. Systematic evaluation revealed that increased crosslinking density enhanced hydrogel hardness in linear progression but compromised stretchability. Although ELP incorporation produced similar stiffening effects, its impact on extensibility reduction was significantly attenuated compared to crosslinking-mediated changes. Through linear superposition analysis of these dual mechanisms, we selected testable formulations with optimal solubility for mechanical characterization (Supplementary Figure S2). The final optimized formulation, featured in the main text, was determined based on matching both Young’s modulus to mucosal benchmarks and extensibility to biological tissue parameters.

**FIGURE 1:**
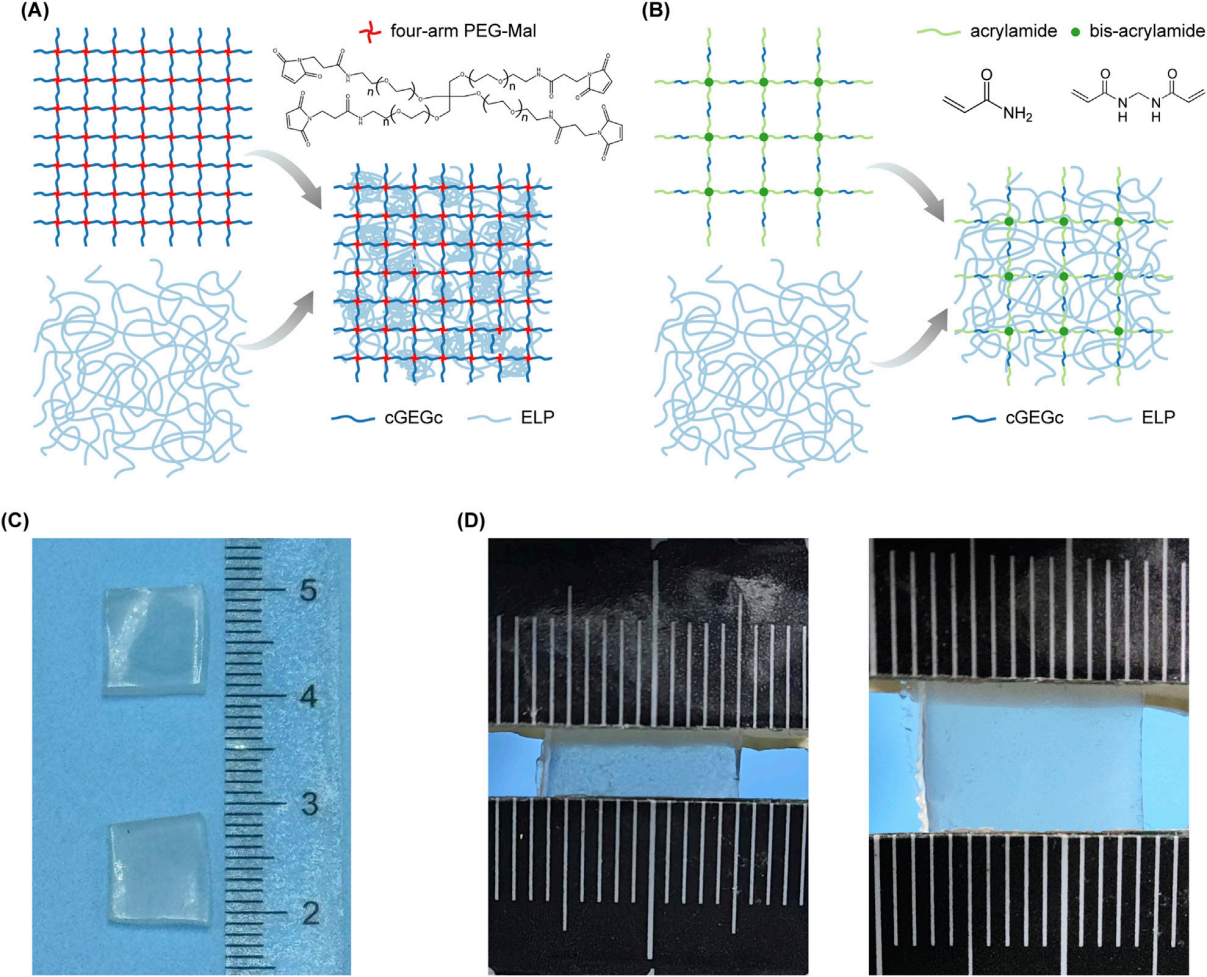
Schematic illustrations of protein-based double-network hydrogels mimicking the properties of oral mucosa. **(A)** Design schematic of the network structure of Palatal PD-Gel. **(B)** Design schematic of the network structure of Buccal PD-Gel. **(C)** Photograph of two hydrogel samples for mechanical testing: Top: Buccal PD-Gel; Bottom: Palatal PD-Gel. **(D)** Photograph of stretching a Palatal PD-Gel.

The harder hydrogel, Palatal PD-Gel, was formulated to emulate the stiff, load-bearing nature of the hard palate mucosa, which endures repetitive compressive forces during mastication due to its keratinized epithelium and dense collagenous stroma. As shown in Figure 1A, the Palatal PD-Gel establishes a dense primary covalently crosslinked network through thiol-maleimide conjugation between cysteine residues at the termini of the engineered elastin-like polypeptide (ELP) variant (cys-GB1-ELP-GB1-cys, cGEGc) and maleimide-functionalized polyethylene glycol (PEG). The network replicates the high stiffness and structural integrity of collagen fibers in masticatory mucosa. Incorporated ELPs undergo a temperature-responsive conformational transition from a soluble state to hydrophobic-dominated aggregates at elevated temperatures, forming a physically entangled secondary network. The ELP sequence was rationally designed to ensure a phase transition temperature below ambient condition (Supplementary Figure S3). The hierarchical architecture—where the secondary ELP network occupies interstitial spaces within the dense primary matrix—mimics the stratified topology of keratinized oral epithelium. This design enhances stress distribution, reducing localized strain by and preventing brittle fracture under cyclic compression, while maintaining energy dissipation capacity that mirrors the rigidity-elasticity equilibrium of natural hard palate tissue.

In contrast, the softer hydrogel, Buccal PD-Gel, was designed to mimic the pliable and deformable buccal mucosa, which features a non-keratinized epithelium and loosely organized connective tissue to accommodate stretching during speech and mastication. Here, acrylamide served as the primary matrix due to its capacity to form flexible, low-modulus networks. The cGEGc protein was integrated into the network formed by acrylamide and bis-acrylamide via thiol-ene reactions, generating a loosely structured hydrogel capable of large, reversible deformations (Figure 1B). A secondary ELP network (without cysteine termini) interpenetrated the acrylamide matrix. The disordered and porous architecture of the acrylamide primary network enables interpenetration of the secondary ELP network, recapitulating the stochastic interweaving of collagen and elastic fiber architectures characteristic of native mucosal lamina propria. This configuration ensures dynamic adaptability, enabling the hydrogel to conform to physiological movements without permanent structural failure, akin to the buccal mucosa’s ability to recover from repeated deformation.

Both hydrogels feature a double-network architecture inspired by the hierarchical organization of oral mucosa. This interpenetrating network structure enhanced the hydrogels’ mechanical performance by facilitating more uniform stress distribution throughout the material (Kim et al., 2021). The primary networks of the composite hydrogels exhibit distinct structural characteristics. In the PEG-based system, the high crosslink density confines the ELP arrangement, resulting in a compressed lamellar morphology. Conversely, in the acrylamide network, the ELP components form an interpenetrating architecture due to their inherent conformational flexibility. This structural dichotomy arises from the fundamental differences in polymer chain mobility and network confinement effects between the two systems. By strategically pairing cGEGc (for covalent crosslinking) with non-reactive ELP (for physical entanglement), we replicated the mechanical properties differences of oral tissues (Supplementary Figure S4). The PEG-cGEGc stiff hydrogel aligns with the high-stiffness requirements of load-bearing regions, while the acrylamide-cGEGc soft hydrogel mirrors the pliability of dynamic mucosal surfaces.

### 3.2 Mechanical characterization of oral mucosa and biomimetic hydrogels

The biomimetic performance of Palatal PD-Gel (mimicking hard palate) and Buccal PD-Gel (mimicking buccal mucosa) was validated through systematic comparison with fresh porcine oral mucosal tissues, selected for their anatomical similarity to human counterparts. Dimensional measurements confirmed an average tissue thickness of ∼1 mm, prompting fabrication of all mucosal samples and hydrogel materials into standardized 10 mm * 10 mm * 1 mm geometries to eliminate scaling artifacts (Figures 1C,D). This dimensional consistency ensured physiologically relevant mechanical comparisons between natural and engineered systems (Supplementary Figure S5).

**FIGURE 2:**
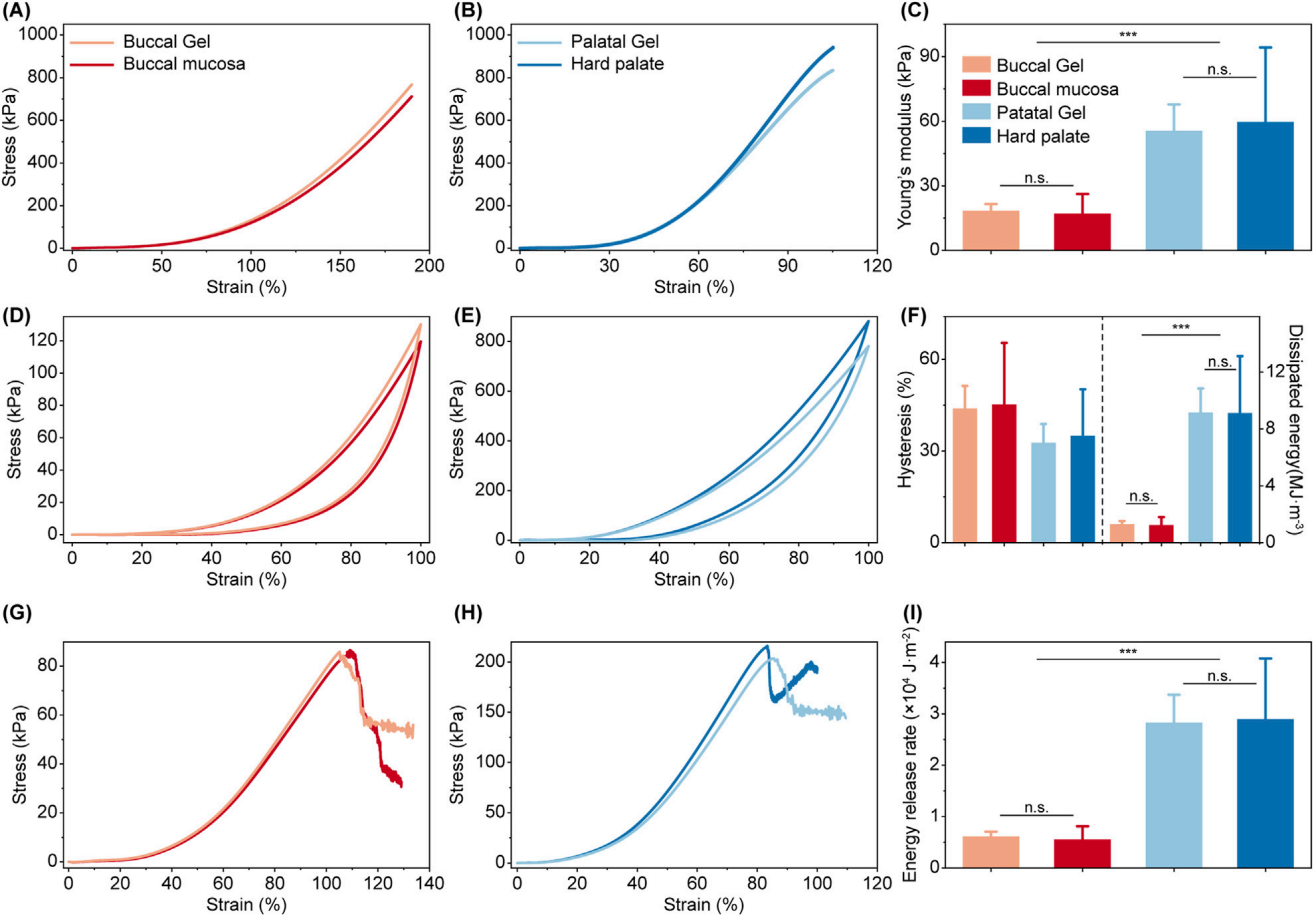
Comparison of mechanical properties between the PD-gels and oral mucosa. **(A, B)** The stress-strain curves of uniaxial cutless stretching of Buccal Gel **(A)** and Palatal Gel **(B)** with corresponding oral mucosa until break. **(C)** Young’s modulus of oral mucosa and biomimetic materials. **(D–E)** Representative stretching-relaxation curves for the Buccal Gel **(D)** and Palatal Gel **(E)** with corresponding oral mucosa. **(F)** Hysteresis of oral mucosa and biomimetic materials. **(G–H)** The stress-strain curves for the precut Buccal Gel **(G)** and Palatal Gel **(H)** with corresponding oral mucosa until break. **(I)** Energy release rate of oral mucosa and biomimetic materials. Statistical significance was assessed using two-tailed Student’s t-test. *: p < 0.05; **: p < 0.01; ***: p < 0.001; n. s.: not significant.

Typical stress-strain curves of the hard palate mucosa, the Palatal PD-Gel, the buccal mucosa, and the Buccal PD-Gel are shown in Figures 2A–C, respectively. The hard palate mucosa can be extended to its original length without rupture with a Young’s modulus of ∼60 kPa at 10% strain, a property derived from its keratinized epithelium and collagen-dense matrix to withstand compressive masticatory forces. The Palatal PD-Gel replicated this modulus (∼58 kPa) through a PEG-cGEGc double-network combining covalent crosslinking for rigidity and elastin-like polypeptide (ELP) entanglement for fracture resistance (Figure 2A). Conversely, the buccal mucosa can be stretched to more than twice its original length without rupture with a lower Young’s modulus of ∼17 kPa at 10% strain, due to its non-keratinized, elastin-rich connective tissue prioritizing flexibility. The Buccal PD-Gel replicates this characteristic through its acrylamide-cGEGc architecture, exhibiting a Young’s modulus of ∼18 kPa while achieving comparable strain capacity (Figure 2B). While both hydrogels diverged in strain-stiffening behavior at high deformations (Supplementary Figure S6), their low-strain mechanical responses closely matched natural tissues, confirming functional emulation of mucosa-specific stress adaptation (Figure 2C).

To further confirm the viscoelastic properties of the biomimetic material, we performed the load/unload cyclic test on all four materials to the same strain (Figures 2D–F). While the buccal mucosa exhibited the highest hysteresis (∼45%) with ∼1.25 MJ/m^3^ dissipated energy, this was attributed to its elastin-mediated viscoelastic behavior that allows gradual stress relaxation during cyclic deformation. The Buccal PD-Gel closely replicated this property (∼44% hysteresis, ∼1.30 MJ/m^3^) through time-dependent acrylamide chain reorientation (Figure 2E). In contrast, the hard palate mucosa showed relatively lower hysteresis (∼35%) with ∼9.12 MJ/m^3^ dissipated energy, reflecting its collagen-dominated microstructure that prioritizes elastic energy storage for structural integrity. The Palatal PD-Gel achieved comparable performance (∼33% hysteresis, ∼9.15 MJ/m^3^) via reversible ELP network reconfiguration (Figure 2D). This energy management strategy in hard palate mucosa protects underlying bone structures during prolonged mastication. The buccal mucosa’s energy-efficient recovery supports sustained functionality during speech and food manipulation. Both hydrogels maintained mechanical responses consistent with biological benchmarks across cyclic loading conditions.

Next, we measured the fracture toughness properties of the hydrogels upon precut notch tensile following the test procedures reported by Zhang et al. (Zhang et al., 2018). The hard palate mucosa has ∼28,900 J/m^2^ fracture energy, which has superior crack resistance (Figure 2G), stemming from its dense collagen matrix and keratinized surface layer that collectively resist crack propagation under cyclic compressive loads. Through ELP-mediated stress redistribution, the Palatal PD-Gel’s fracture energy is ∼28,300 J/m^2^, which perfectly corresponded to the anti-fracture property of the hard palate mucosa. On other hand, the buccal mucosa has lower fracture energy (∼5560 J/m^2^, Figure 2H), resulting from its elastin-rich extracellular matrix that permits compliant deformation during facial articulation while preventing tissue rupture. Through acrylamide matrix ductility the Buccal PD-Gel’s fracture energy was matched ∼6140 J/m^2^. These measurements confirmed that both synthetic materials preserved the fracture resistance characteristics of their biological counterparts (Figure 2I).

The molecular design strategy successfully bridged microscale network interactions (PEG/acrylamide crosslinking density, ELP phase distribution) with macroscale mechanical parity. By preserving structural stability while replicating mucosa-specific energy dissipation and fracture mechanics, these hydrogels establish a platform for engineering oral tissue constructs that address mechanical heterogeneity. This advancement enables next-generation mucosal repair therapies with physiologically adaptive performance.

### 3.3 Polymer network and surface hydrophilicity of oral mucosa and biomimetic hydrogels

**FIGURE 3:**
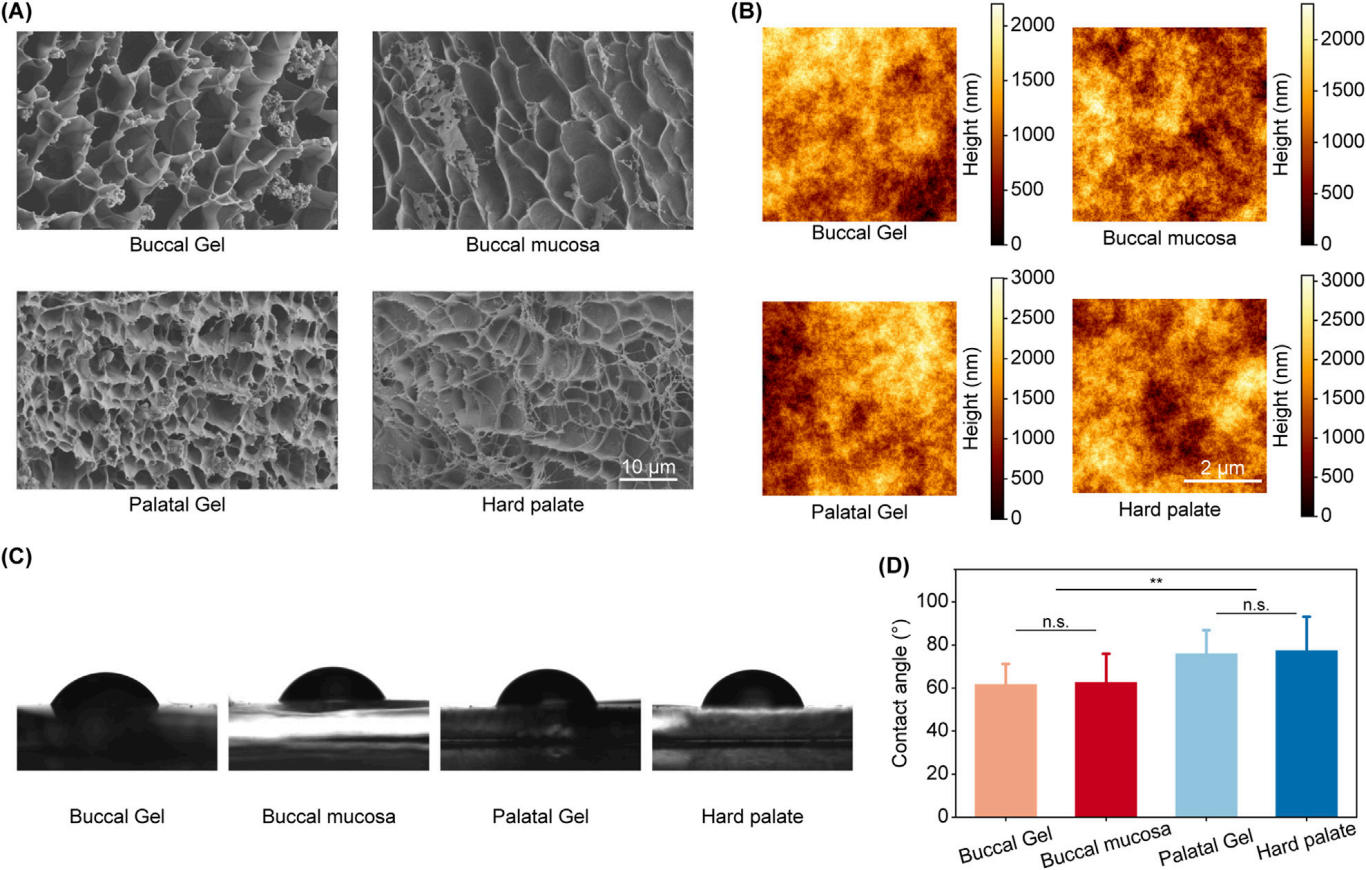
Comparison of surface characterization between biomimetic hydrogels and oral mucosa. **(A)** SEM images of biomimetic materials and oral mucosa. **(B)** AFM scanning maps of biomimetic materials and oral mucosa. **(C)** Representative images of contact angle measurements. **(D)** Statistical analysis of contact angles. Statistical significance was assessed using two-tailed Student’s t-test. *: p < 0.05; **: p < 0.01; ***: p < 0.001; n. s.: not significant.

To systematically evaluate the microstructural congruence between biomimetic materials and native mucosal tissues, a suite of physical characterizations was conducted as systematically benchmarked. Comparative scanning electron microscopy (SEM) microstructural analysis demonstrated that hydrogel analogues precisely replicated the three-dimensional fibrillar organization characteristic of native biological specimens, as evidenced by congruent polymer network architectures at submicron resolution (Figure 3A). Notably, high-resolution SEM imaging revealed critical distinctions in elastin distribution patterns. In both the buccal mucosa and its hydrogel counterpart, elastin exhibited interstitial dispersion within the fibrillar matrix, whereas hard palate specimens and Palatal PD-Gel demonstrated elastin’s peripheral alignment along collagen bundles. The supramolecular structural difference, mediated through fiber network-elastin interfacial dynamics, mechanistically governs the contrasting viscoelastic profiles: the former permitting flexibility and extensibility and the latter enforcing structural rigidity. These similarities in network structure and porosity enable the hydrogels to accurately simulate the mechanical and barrier properties of oral mucosa, providing a reliable platform for studying interactions between oral tissues and foreign substances.

Subsequently, we performed detailed surface flatness assessments to compare the smoothness of the biomimetic hydrogels with that of natural oral mucosa. Atomic force microscopy (AFM) measurements revealed that the hydrogels exhibited a surface smoothness closely aligned with native tissue, minimizing surface irregularities that could compromise barrier function or promote the accumulation of external particles (Figure 3B). This similarity in surface texture is essential for ensuring that the hydrogels perform effectively in dynamic oral environments, maintaining cleanliness and reducing the risk of microbial adhesion or biofilm formation.

In addition, the hydrophilicity of the hydrogels—an important factor influencing wettability and interaction with biological fluids—was evaluated through contact angle measurements. The results showed that the hydrogels’ contact angles closely resembled those of natural oral mucosa, indicating similar surface energy characteristics (Figures 3C,D). This hydrophilic nature is critical for facilitating interactions with aqueous environments, allowing the hydrogels to integrate seamlessly within biological systems. Furthermore, their ability to retain moisture enhances their capacity to mimic the moist environment of oral tissues, which is vital for maintaining tissue homeostasis and promoting cell adhesion.

### 3.4 Penetration dynamics of nanoparticles in natural and biomimetic oral mucosa

The particulate permeability of oral mucosa constitutes a dynamic interface governing barrier-protection and substance transport. Recent findings reveal intact mucosa exhibits size-selective exclusion, with less than 5% penetration efficiency for particles exceeding 80 nm (Xu et al., 2023). Breakthroughs in nanomedicine include pH-responsive carriers achieving 12-fold enhanced permeation rates in buccal drug delivery systems (Guo et al., 2023). Clinically, engineered nanoparticles have gained clinical traction for mucoadhesion-based respiratory therapies (Ensign et al., 2012). This evidence highlights mucosal particle adhesion capacity as a critical parameter requiring optimization in biomimetic mucosal interfaces.

**FIGURE 4:**
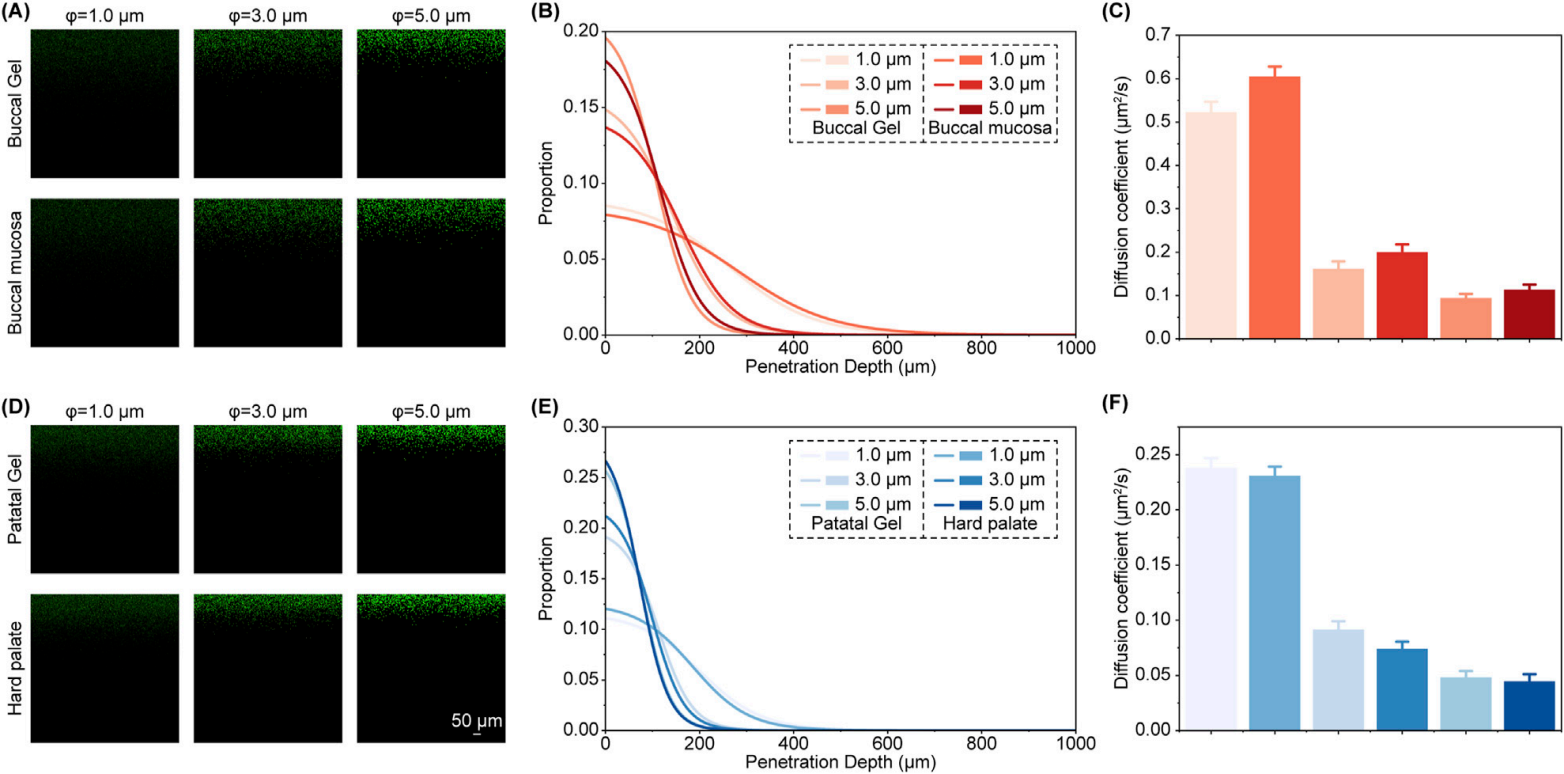
Diffusion behavior of fluorescent particles with different particle sizes. **(A–B)** Fluorescence images showing the diffusion of particles of different sizes in the buccal mucosa and soft hydrogel after 8 h **(A)**, along with the corresponding distribution statistics **(B)**. The fluorescence images for each hour are shown in Supplementary Figures S7, S8. **(C)** Diffusion coefficients of particles in the buccal mucosa and soft hydrogel. **(D–E)** Fluorescence images showing the diffusion of particles of different sizes in the hard palate and rigid hydrogel after 8 h **(C)**, along with the corresponding distribution statistics **(D)**. The fluorescence images for each hour are shown in Supplementary Figures S9–S10. **(F)** Diffusion coefficients of particles in the hard palate and rigid hydrogel.

To examine the penetration behavior of foreign particulate matter across various mucosal tissues, we performed a series of experiments using fluorescent particles with diameters of 1 μm, 3 μm, and 5 μm. These particle sizes were chosen to represent a range of airborne and environmental contaminants that oral tissues encounter, providing insight into the tissue’s selective barrier properties (Figure 4; Supplementary Figures S7–S10). The fluorescent labeling enabled precise tracking of particle distribution within both the natural mucosal tissues and the engineered hydrogels, allowing for detailed visualization and statistical analysis of particle behavior over time.

The results show that the buccal mucosa’s non-keratinized stratified epithelium and loose lamina propria exhibited structural homology with the Buccal PD-Gel’s tunable polymer network. This architectural congruence enabled analogous particle permeation profiles: 1 μm particles demonstrated significantly deeper penetration in both native tissue and synthetic analogue compared to larger particulates, while 5 μm particles remained predominantly surface-bound with minimal permeation depth in both systems (Figure 4A). Intermediate-sized 3 μm particles displayed penetration characteristics proportionally between the smaller and larger test particles, with near-complete concordance observed between the two distinct particulate systems through comparative measurements (Figure 4B). We analyzed particle distribution patterns through the given model, revealing that diffusion velocity exhibited a negative correlation with particle diameter, consistent with theoretical predictions (Figure 4C). Notably, prolonged testing revealed significantly retarded penetration progression for 5 μm particles at greater depths, with 3 μm particles also showing deceleration (Supplementary Figures S7–S8). This suggests that the denser internal network structure of the material, characterized by smaller interstitial spaces compared to the surface layer, impedes penetration of larger particulates. In contrast, 1 μm particles maintained unimpeded permeation throughout the observation period.

On the other hand, the hard palate mucosa’s elevated elastic modulus and reduced permeability originate from its densely packed collagen fibrillar architecture. In the Palatal PD-Gel, short-chain PEG networks achieved biomimetic particle confinement, with 3–5 μm particles demonstrating equivalent surface retention hierarchies between biological and synthetic systems (Figures 4D,E). Both systems maintained matched penetration profiles for 1 μm particles, confirming preserved nanoscale permeability while replicating microscale barrier functionality. Application of the given model to this system similarly demonstrated diameter-dependent diffusion velocities with an inverse size-velocity relationship (Figure 4F). The overall permeation rates were notably lower than those observed in both buccal mucosa and Buccal PD-Gel systems, aligning with expected behavior based on structural differences. Extended observation periods revealed that larger particles became arrested at shallower penetration depths compared to earlier stages (Supplementary Figures S9–S10). The observed size-exclusion threshold for particulates beyond functional transport dimensions arises from structural congruence between the high-density polymeric matrix and native mucosal architecture, mediated through elastin-mediated interfibrillar space occupation within the supramolecular network.

### 3.5 Biocompatibility of biomimetic hydrogels for oral epithelial cell growth

**FIGURE 5:**
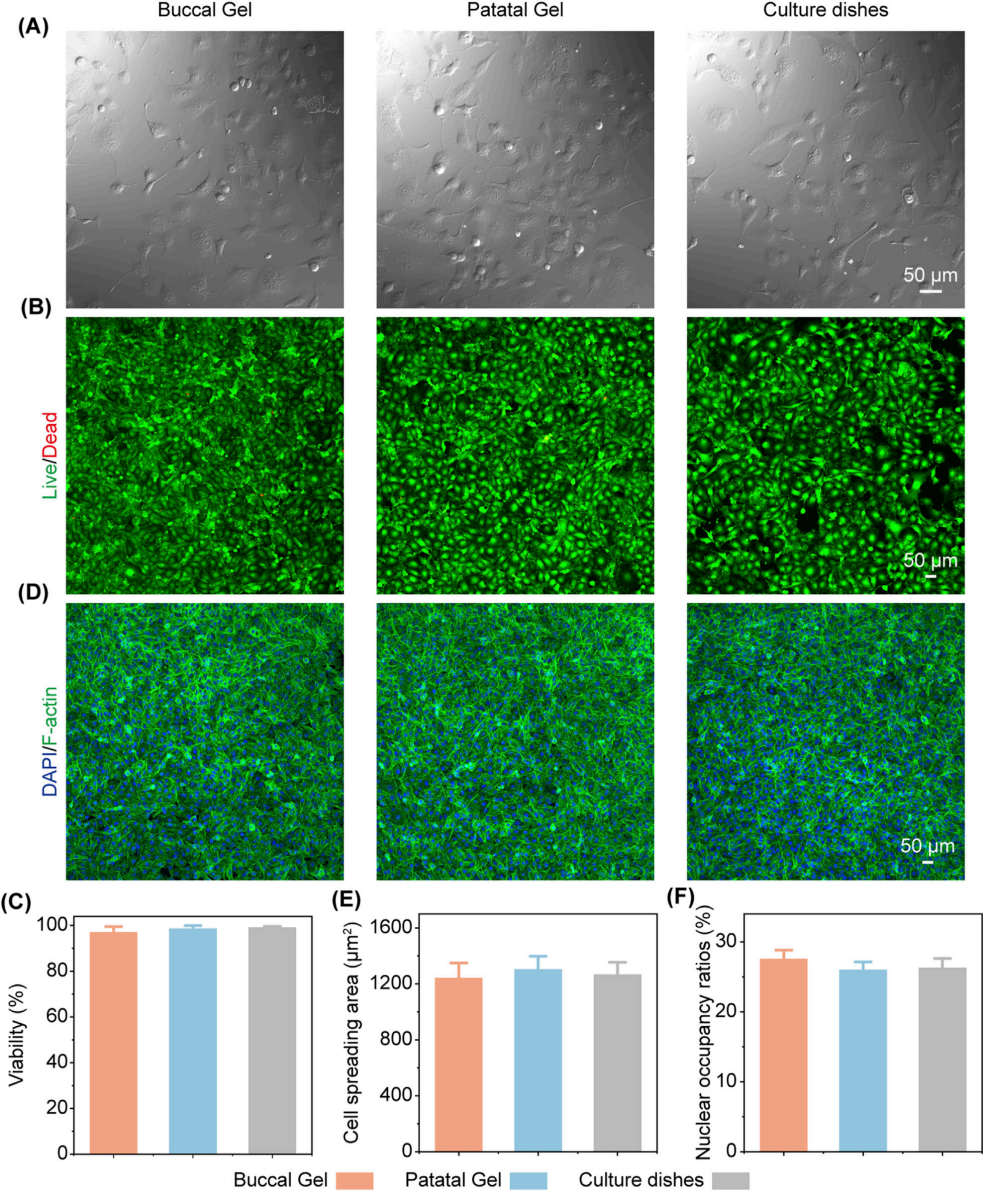
Evaluation of epithelial cell culture on gels *versus* cell culture dishes. **(A)** Bright-field images of epithelial cells cultured on gels and cell culture dishes. **(B)** Live/dead staining of epithelial cells on corresponding substrates (green: live cells, red: dead cells). **(C)** Quantification of cell viability showing comparative survival rates on gels and conventional culture dishes. **(D)** Immunofluorescence images of epithelial cells stained with DAPI (blue, nuclei) and phalloidin (green, F-actin) on corresponding substrates. **(E–F)** Statistical analysis of cell spreading area **(E)** and nucleus-to-cytoplasm area ratio **(F)**.

To evaluate the biocompatibility of the engineered hydrogels, we conducted a series of *in vitro* experiments by culturing human oral epithelial cells directly onto the hydrogel surfaces as well as on conventional cell culture dishes as a control group. These experiments aimed to assess whether the hydrogels could support cell adhesion, proliferation, and survival, which are critical factors for materials intended to function as substitutes for native tissues. A live-dead staining assay was employed to measure the viability of the cultured cells quantitatively and qualitatively. This assay uses fluorescent dyes to differentiate between living and dead cells, providing a reliable measure of cell health and biocompatibility over time. The experimental results clearly demonstrated that the human epithelial cells adhered effectively to both hydrogel formulations and control dishes, spreading uniformly across the surfaces and exhibiting typical epithelial morphology (Figure 5A). The live cells, stained with green fluorescence, were abundant throughout the hydrogel matrices and control substrates, while dead cells, stained with red fluorescence, were sparse in all groups, indicating a high level of cell viability (Figure 5B). Quantitative analysis of the staining data confirmed that both hydrogel types achieved a cell survival rate of over 95%, comparable to the viability observed in conventional culture dishes, reflecting the material’s ability to sustain a favorable microenvironment for cell growth (Figure 5C).

Furthermore, we conducted comparative analyses of cellular spreading behavior and morphology across both hydrogel formulations and control culture dishes. Under identical initial seeding densities, cells cultured on the hydrogels demonstrated spreading patterns and morphological characteristics closely resembling those on the culture dishes. Quantitative measurements of spreading area revealed average values of ∼1,240 μm^2^ and ∼1,300 μm^2^ for hydrogel type A and B respectively, statistically equivalent to the ∼1,270 μm^2^ observed in control dishes. Nuclear area analysis through DAPI staining showed comparable nuclear occupancy ratios of ∼28% and ∼26% for hydrogel-cultured cells *versus* ∼26% for dish-cultured controls. These data confirm that the engineered hydrogels permit normal cellular spreading dynamics without imposing mechanical constraints or biochemical interference on epithelial cell behavior.

## 4 Discussion

The oral mucosa serves as the first line of defense in the upper respiratory tract, playing a critical role in protecting against external physical, chemical, and biological agents (Şenel, 2021). Many excellent *in vitro* oral mucosal models have been developed, including reconstructed epithelium (Schmalz et al., 2000; Thurnheer et al., 2014; Bostanci et al., 2015; Hu et al., 2022; Machla et al., 2024), connective tissue (Makkar et al., 2022), full-thickness gingival equivalents (Moharamzadeh et al., 2008; Moharamzadeh et al., 2009; Buskermolen et al., 2018; Shang et al., 2018; Ingendoh-Tsakmakidis et al., 2019), and biofabricated full-thickness gingiva-on-chip (Muniraj et al., 2023). However, most of the models use oral keratinocytes to form tissues. While they can provide reliable toxicity testing results, they are often complex, significantly increasing both time and economic costs in high-throughput testing. In this study, we shift the focus towards the macro-scale mechanical properties and micro-scale network structures to develop two types of hydrogel materials that accurately simulate the distinct characteristics of oral mucosa from different regions of the mouth.

To achieve this, it was essential to measure and analyze the mechanical properties of real oral mucosa tissue. We prioritized the replication of the mechanical properties, not only because the macroscopic properties of hydrogels are determined by their microscopic network structure, and materials with similar mechanical properties are likely to have comparable microscopic structures (Vedadghavami et al., 2017), but more importantly, the closeness of these mechanical properties directly determines whether the material can serve as a biomimetic substitute for real biological tissue. Many studies have shown that numerous behaviors in biological tissues, especially cellular behaviors, are directly influenced and regulated by the mechanical properties of the surrounding tissue (Yang et al.; Chan and Odde, 2008; Roca-Cusachs et al., 2017; Yang et al., 2021). Although some existing studies have reported on the mechanical behavior of human oral mucosa (Choi et al., 2020a), these samples underwent chemical fixation before testing, which is known to significantly alter the material’s native properties (Verstraete et al., 2015). Additionally, ethical and logistical constraints limit access to fresh human oral mucosa for direct testing. Therefore, we used porcine oral mucosa, which shares similar structure and function with human tissue, as a substitute for testing (Wernersson et al., 2005; Walters and Prather, 2013).

We collected and tested mucosa samples from two different regions of the porcine oral cavity to establish benchmarks for designing biomimetic hydrogel materials. Our results show that the stiffness of the hydrogels closely matches that of the native oral mucosa. Moreover, further characterization of the micro-scale network structure and hydrophilic/hydrophobic properties revealed high similarity between the hydrogels and real tissues, indicating successful structural mimicry.

From a mechanistic perspective, the hierarchical network architecture of our hydrogels—comprising covalent crosslinks and ELP-mediated physical entanglements—mimics the dynamic reciprocity between collagen fibrils and elastin microfibrils in native mucosa. The primary covalent network (PEG or acrylamide) provides structural integrity akin to collagen’s role, while the secondary ELP network replicates elastin’s energy dissipation. Such molecular-level orchestration establishes a robust framework for designing biomimetic polymer networks.

The particle permeation experiments confirmed that the hydrogel materials effectively simulate the barrier function of oral mucosa, resisting the penetration of foreign particles. These findings suggest that the hydrogels can serve as reliable substitutes for oral mucosa in various experimental scenarios, including studies on drug delivery and pathogen interactions. Additionally, biocompatibility tests demonstrated that oral mucosal cells could adhere to and grow on the hydrogel surfaces, confirming the potential of these materials for future cell-based studies.

However, our functional validation has certain limitations. While the hydrogel’s size-selective permeability to micron-scale particles aligns with native mucosa, the current system does not account for the dynamic biochemical interactions of mucin glycoproteins, which critically influence particle adhesion and transport kinetics *in vivo*. Furthermore, the static mechanical testing paradigm neglects the impact of cyclic physiological forces (e.g., tongue movement or mastication) on hydrogel durability—a crucial factor for long-term mucosal substitutes. Future studies should incorporate mucin-functionalized networks and dynamic mechanical loading to better replicate the mucosa’s tribological behavior.

Beyond the scope of oral mucosa simulation, the design principles and methodologies used in this study can serve as a foundation for the development of biomimetic materials for other mucosal tissues. Mucosal surfaces in different organs, such as the respiratory tract, gastrointestinal system, and urogenital tract, possess distinct mechanical, structural, and functional properties essential for their specific roles. For example, respiratory mucosa requires both elasticity and mucociliary clearance capability, while intestinal mucosa must balance selective permeability with a robust barrier function (Jankowski et al., 1994; Huff et al., 2019). By customizing hydrogel formulations according to these tissue-specific requirements, researchers can develop materials that closely replicate the behavior of different mucosae, facilitating studies in drug delivery, toxicology, and disease modeling.

The application of biomimetic hydrogels extends beyond fundamental research. These materials hold promise for regenerative medicine, where they could serve as scaffolds for tissue repair or as platforms for personalized drug delivery systems (Sun et al., 2024; Zhang et al., 2024). Future work could further enhance these materials by integrating bioactive components or designing dynamic, stimuli-responsive structures to better mimic the behavior of living tissues.

In summary, our study not only demonstrates the successful fabrication of hydrogel materials that closely mimic the mechanical and structural properties of oral mucosa but also provides a roadmap for expanding this approach to other mucosal tissues. This work bridges material science and biomedical applications, offering new opportunities for advancing research in pharmacology, toxicology, tissue engineering, and personalized medicine.

## Data Availability

The datasets presented in this study can be found in online repositories. The names of the repository/repositories and accession number(s) can be found in the article/Supplementary Material.
