# Supplementary material for: Protein-based double-network hydrogels mimicking oral mucosa
Source: Front Chem. 2025 Jun 13;13:1618870. doi: 10.3389/fchem.2025.1618870 (PMC12202402; doi:10.3389/fchem.2025.1618870)
Supplement: Supplementary file 1 [file DataSheet1.docx]

Supplementary Material

# Supplementary Data

## Protein sequence

Cys-GB1-ELP-GB1-Cys
MRGSHHHHHHCGSMDTYKLILNGKTLKGETTTEAVDAATAEKVFKQYANDNGVDGEWTYDDATKTFTVTERSVPGVGVPGVGVPGEGVPGVGVPGVGVPGVGVPGVGVPGEGVPGGLRSVPGVGVPGVGVPGEGVPGVGVPGVGVPGVGVPGVGVPGEGVPGGLRSMDTYKLILNGKTLKGETTTEAVDAATAEKVFKQYANDNGVDGEWTYDDATKTFTVTERSGTC

ELP
MRGSHHHHHHGSMDTYKLILNGKTLKGETTTEAVDAATAEKVFKQYANDNGVDGEWTYDDATKTFTVTERSVPGVGVPGVGVPGEGVPGVGVPGVGVPGVGVPGVGVPGEGVPGGLRSVPGVGVPGVGVPGEGVPGVGVPGVGVPGVGVPGVGVPGEGVPGGLRSVPGVGVPGVGVPGEGVPGVGVPGVGVPGVGVPGVGVPGEGVPGGLRSVPGVGVPGVGVPGEGVPGVGVPGVGVPGVGVPGVGVPGEGVPGGLRSVPGVGVPGVGVPGEGVPGVGVPGVGVPGVGVPGVGVPGEGVPGGLRSVPGVGVPGVGVPGEGVPGVGVPGVGVPGVGVPGVGVPGEGVPGGLRSVPGVGVPGVGVPGEGVPGVGVPGVGVPGVGVPGVGVPGEGVPGGLRSVPGVGVPGVGVPGEGVPGVGVPGVGVPGVGVPGVGVPGEGVPGGLRSVPGVGVPGVGVPGEGVPGVGVPGVGVPGVGVPGVGVPGEGVPGGLRSVPGVGVPGVGVPGEGVPGVGVPGVGVPGVGVPGVGVPGEGVPGGLRSVPGVGVPGVGVPGEGVPGVGVPGVGVPGVGVPGVGVPGEGVPGGLRSVPGVGVPGVGVPGEGVPGVGVPGVGVPGVGVPGVGVPGEGVPGGLRSMDTYKLILNGKTLKGETTTEAVDAATAEKVFKQYANDNGVDGEWTYDDATKTFTVTERS

## Gelation Mechanism

Maleimide-mediated bioconjugation represents a cornerstone methodology in polymer-protein hybrid network formation, owing to its rapid reaction kinetics and high selectivity toward cysteine residues in proteins (Koniev and Wagner, 2015). This specificity enables covalent integration of proteins into polymer matrices via radical-mediated thiol-ene click chemistry, wherein terminal cysteine thiols undergo selective coupling with maleimide-functionalized polymers (Huang et al., 2019). Such mechanisms underpin diverse applications, including targeted therapeutics, protein microarrays, and biomimetic assemblies for studying protein function in biological contexts (Hermanson, 2013).

In parallel, photopolymerizable hydrogel systems employ acrylamide-based monomers and crosslinkers such as N,N’-methylenebisacrylamide (MBAA) to establish three-dimensional networks. Lithium phenyl-2,4,6-trimethylbenzoylphosphinate (LAP), a water-soluble Type I photoinitiator, initiates radical polymerization under visible or UV light (365–400 nm). Upon irradiation, LAP generates free radicals that cleave the double bonds of acrylamide and MBAA monomers, triggering chain propagation and crosslinking. The resulting network incorporates proteins via dual mechanisms: (1) physical entrapment within the polymer matrix and (2) covalent conjugation through residual unsaturated bonds that participate in thiol-ene reactions with cysteine thiols. This synergistic approach ensures structural stability while preserving protein functionality, making it particularly suitable for cell encapsulation and bioactive hydrogel fabrication.

Key advantages of LAP over conventional photoinitiators (e.g., Irgacure 2959) include enhanced water solubility, reduced cytotoxicity at lower concentrations, and compatibility with visible-light activation, thereby improving cell viability during in situ gelation processes.

# Supplementary Figures and Tables

## Supplementary Figures


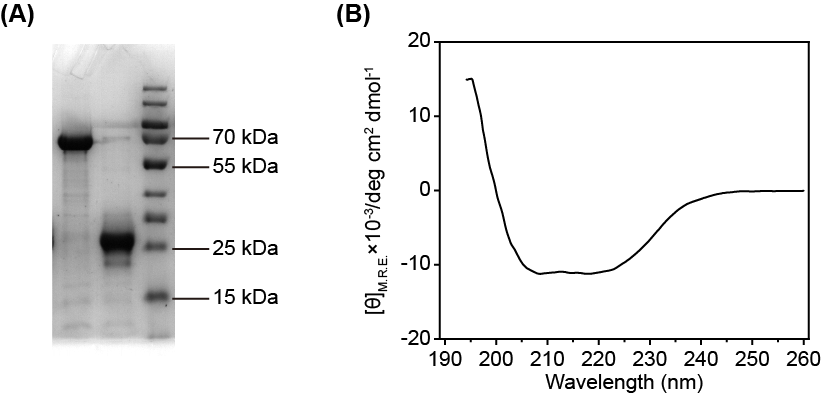


**Supplementary Figure 1.** (A) SDS-PAGE analysis of the cGEGc and ELP proteins. The left lane represents the $\mathrm{ELP}_{12}$ protein (62.53 kDa), the middle lane corresponds to the cGEGc protein (23.93 kDa), and the right lane contains the molecular weight ladder. (B) The Far-UV circular dichroism spectral results of the cGEGc protein demonstrated the proper folding of the GB1 structure.


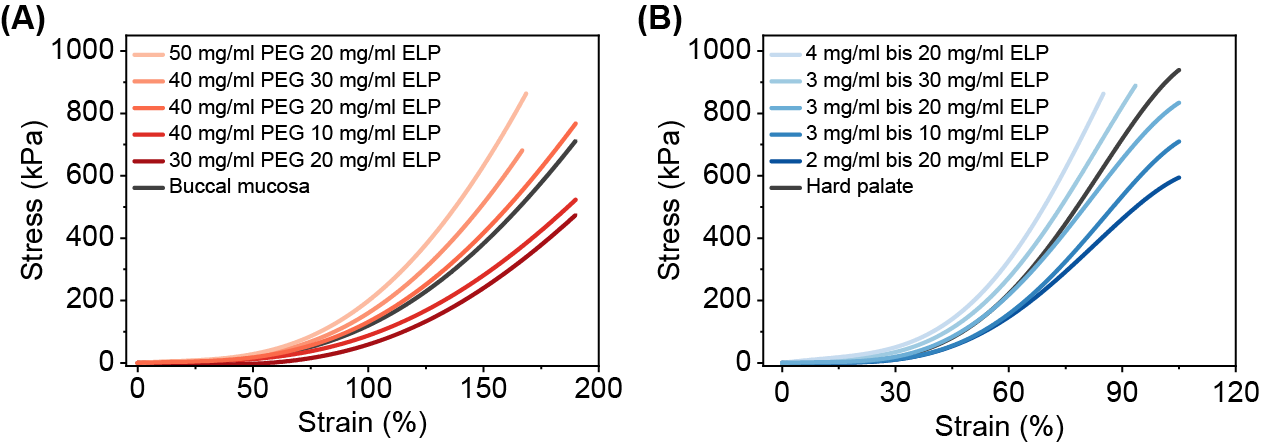


**Supplementary Figure 2.** The stress-strain curves of Buccal PD-Gel (A) and Palatal PD-Gel (B) after adjusting crosslinking density (PEG or bis) and primary/secondary network ratios (ELP), along with their comparative results against corresponding human tissues, demonstrate that the optimized formulation presented in the manuscript achieves the best simulation effect on tissue biomechanical properties. Notably, the cGEGc protein concentration was maintained in excess throughout the experiments, and since its concentration variations won’t influence network formation, thus experimental results regarding its concentration variations were excluded from presentation.


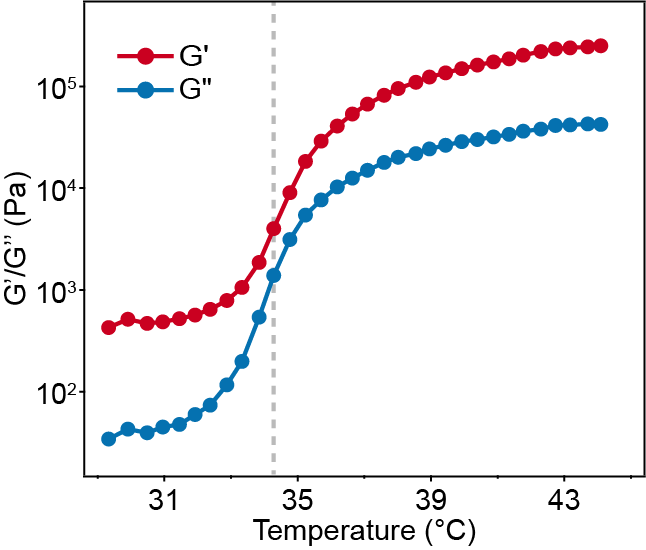


**Supplementary Figure 3.** Temperature-dependent viscoelastic transition of ELP. ELP protein concentration is 20 mg/mL. The gray dashed line indicates that, the lower critical solution temperature (LCST), was experimentally validated through rheological testing to ensure it remained below 37°C (~ 34°C).


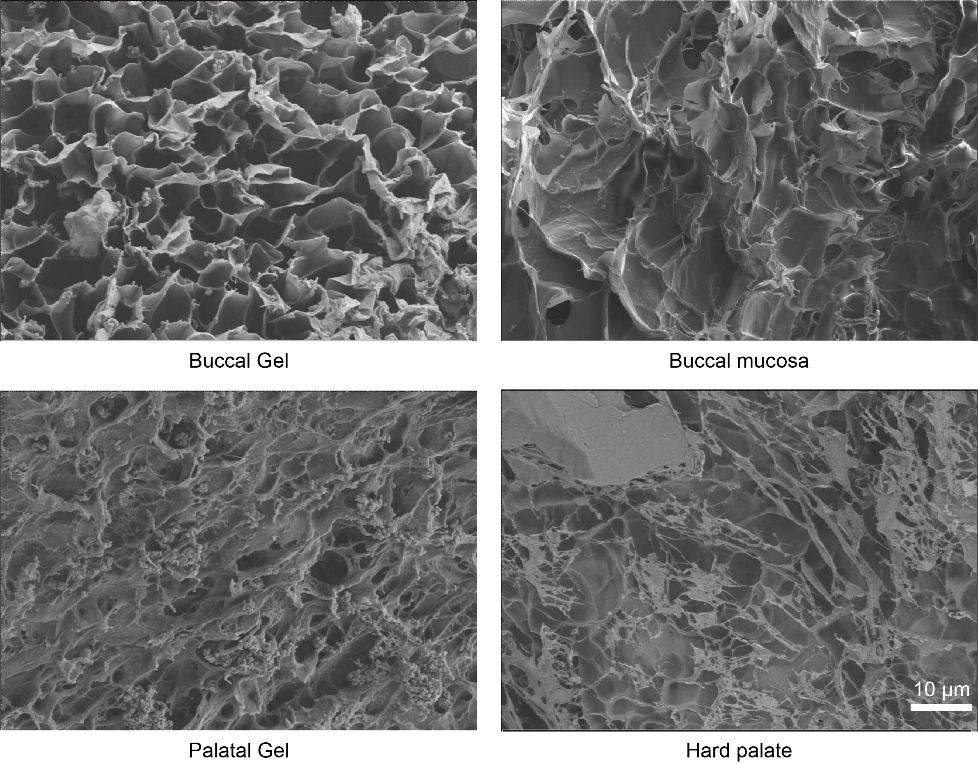


**Supplementary Figure 4.** SEM images of biomimetic materials and oral mucosa.


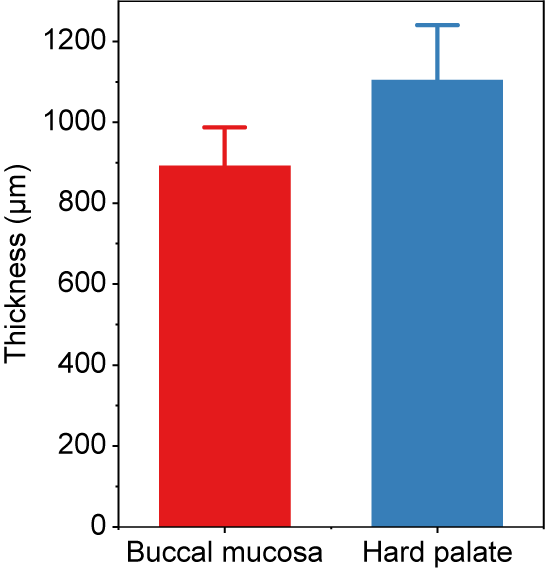


**Supplementary Figure 5.** Statistical Analysis of Oral Mucosa Thickness.


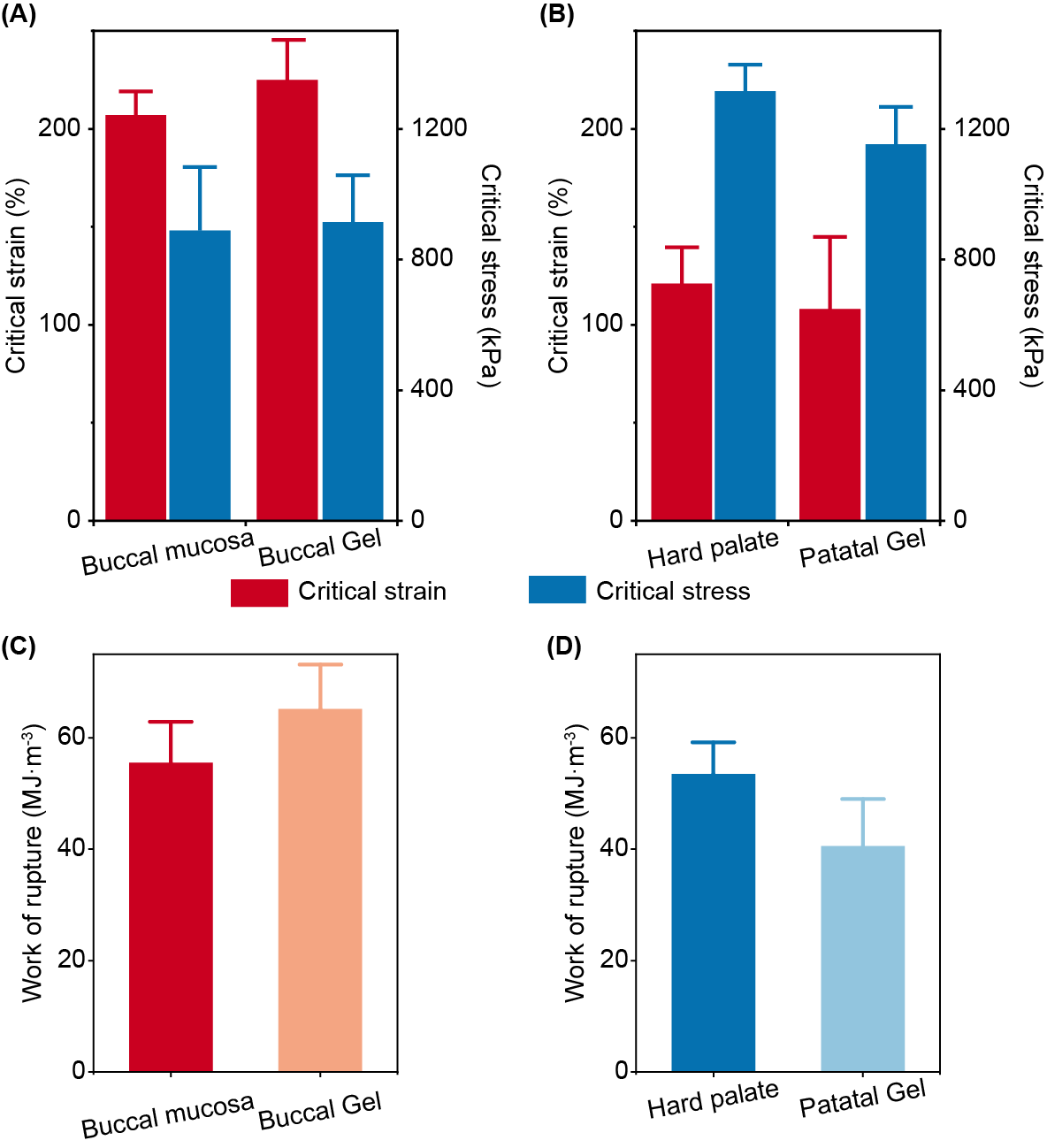


**Supplementary Figure 6.** (A-B) Critical strain and critical stress of hard palate and Palatal Gel (A) and buccal mucosa and Buccal Gel (B). (C-D) Work of rupture of hard palate and Palatal Gel (C) and buccal mucosa and Buccal Gel (D).


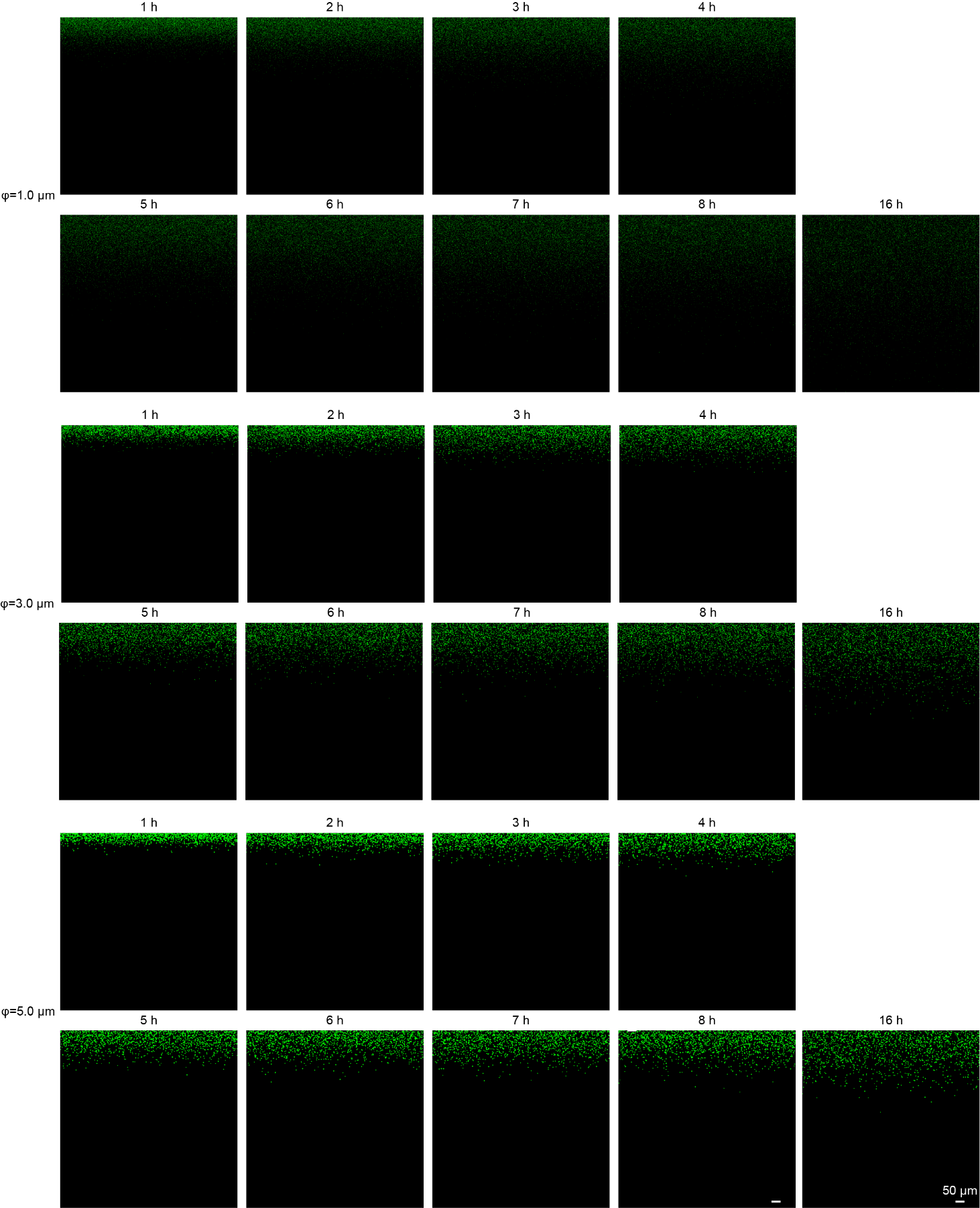


**Supplementary Figure 7.** Fluorescence images showing the diffusion of particles of different sizes in the Buccal Gel at hourly intervals.


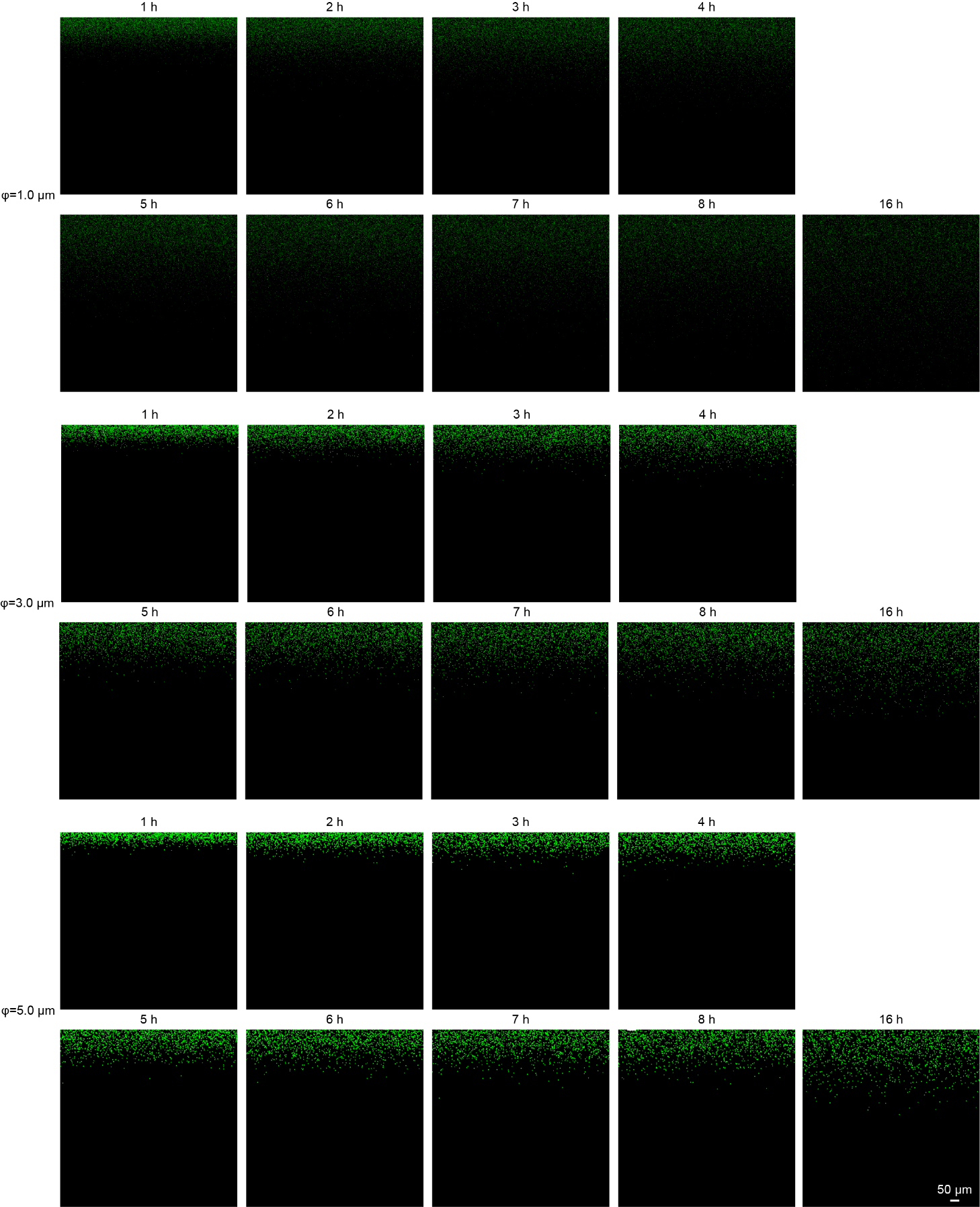


**Supplementary Figure 8.** Fluorescence images showing the diffusion of particles of different sizes in the buccal mucosa at hourly intervals.


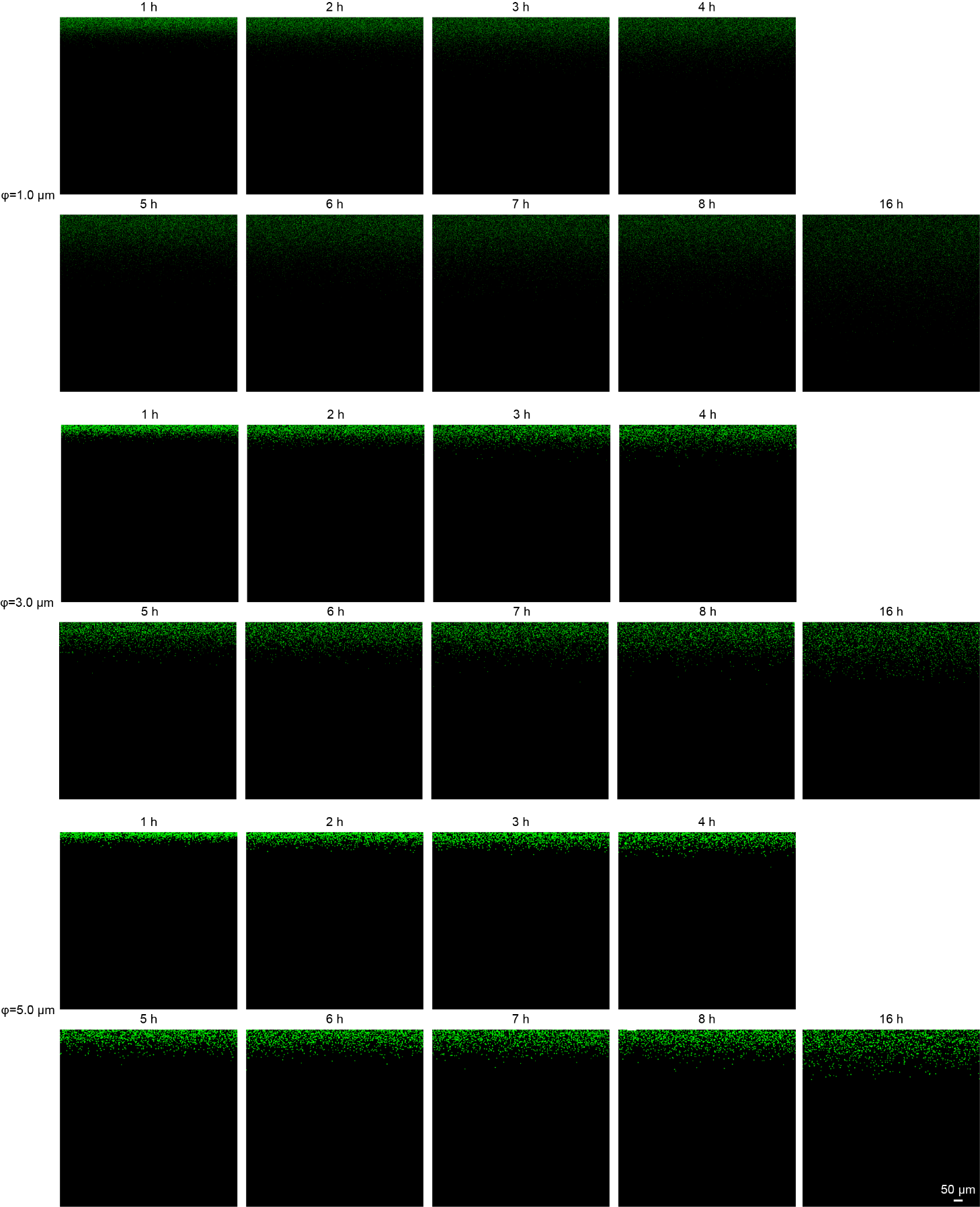


**Supplementary Figure 9.** Fluorescence images showing the diffusion of particles of different sizes in the Palatal Gel at hourly intervals.


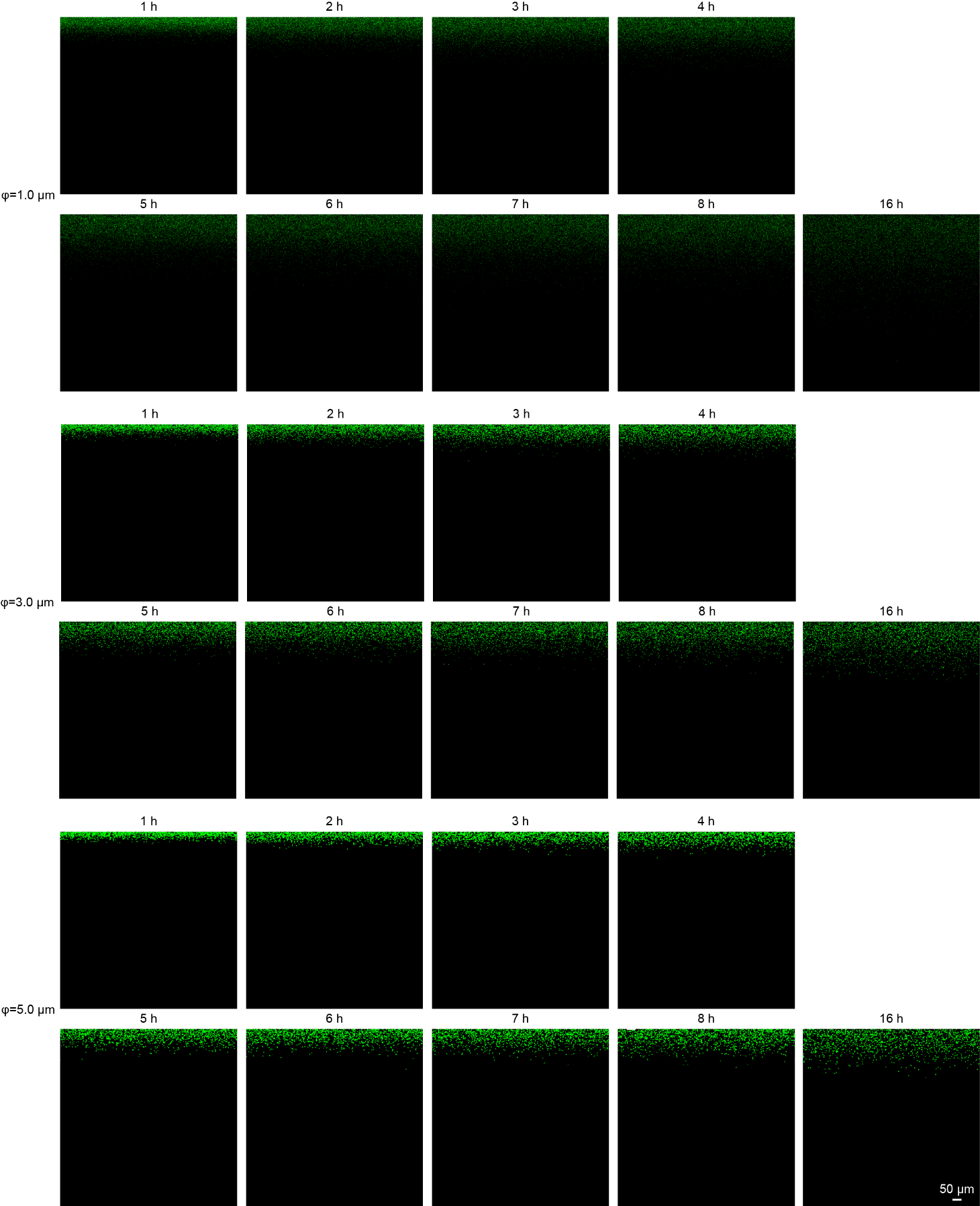


**Supplementary Figure 10.** Fluorescence images showing the diffusion of particles of different sizes in the hard palate at hourly intervals.

**Reference**

Hermanson, G.T. (2013). "Chapter 1 - Introduction to Bioconjugation," in *Bioconjugate Techniques (Third Edition),* ed. G.T. Hermanson. (Boston: Academic Press), 1-125.

Huang, W., Wu, X., Gao, X., Yu, Y., Lei, H., Zhu, Z., et al. (2019). Maleimide–thiol adducts stabilized through stretching. *Nature Chemistry* 11(4)**,** 310-319. doi: 10.1038/s41557-018-0209-2.

Koniev, O., and Wagner, A. (2015). Developments and recent advancements in the field of endogenous amino acid selective bond forming reactions for bioconjugation. *Chemical Society Reviews* 44(15)**,** 5495-5551. doi: 10.1039/C5CS00048C.
